# Associations between red blood cell variants and malaria among children and adults from three areas of Uganda: a prospective cohort study

**DOI:** 10.1186/s12936-020-3105-3

**Published:** 2020-01-15

**Authors:** Elijah Kakande, Bryan Greenhouse, Francis Bajunirwe, Chris Drakeley, Joaniter I. Nankabirwa, Andrew Walakira, Samuel L. Nsobya, Agaba Katureebe, John Rek, Emmanuel Arinaitwe, Philip J. Rosenthal, Moses R. Kamya, Grant Dorsey, Isabel Rodriguez-Barraquer

**Affiliations:** 1 Infectious Diseases Research Collaboration, 2C Nakasero Hill Road, Kampala, Uganda; 2 Department of Medicine, University of California, San Francisco, CA 94110 USA; 3 Department of Community Health, Mbarara University of Science and Technology, Mbarara, Uganda; 4 Immunity and Infection, London School of Hygiene & Tropical Medicine, Keppel Street, London, WC1E 7HT UK

**Keywords:** Red blood cell variants, Erythrocyte, Malaria, Plasmodium, Sickle hemoglobin, Thalassemia

## Abstract

**Background:**

Multiple red blood cell (RBC) variants appear to offer protection against the most severe forms of *Plasmodium falciparum* malaria. Associations between these variants and uncomplicated malaria are less clear.

**Methods:**

Data from a longitudinal cohort study conducted in 3 sub-counties in Uganda was used to quantify associations between three red blood cell variants Hb [AA, AS, S (rs334)], alpha thalassaemia 3.7 kb deletion, and glucose-6-phosphate dehydrogenase deficiency A—(G6PD 202A genotype) and malaria incidence, parasite prevalence, parasite density (a measure of anti-parasite immunity) and body temperature adjusted for parasite density (a measure of anti-disease immunity). All analyses were adjusted for age, average household entomological inoculation rate, and study site. Results for all variants were compared to those for wild type genotypes.

**Results:**

In children, HbAS was associated, compared to wild type, with a lower incidence of malaria (IRR = 0.78, 95% CI 0.66–0.92, p = 0.003), lower parasite density upon infection (PR = 0.66, 95% CI 0.51–0.85, p = 0.001), and lower body temperature for any given parasite density (− 0.13 ℃, 95% CI − 0.21, − 0.05, p = 0.002). In children, HbSS was associated with a lower incidence of malaria (IRR = 0.17, 95% CI 0.04–0.71, p = 0.02) and lower parasite density upon infection (PR = 0.31, 95% CI 0.18–0.54, p < 0.001). α−/αα thalassaemia, was associated with higher parasite prevalence in both children and adults (RR = 1.23, 95% CI 1.06–1.43, p = 0.008 and RR = 1.52, 95% CI 1.04–2.23, p = 0.03, respectively). G6PD deficiency was associated with lower body temperature for any given parasite density only among male hemizygote children (− 0.19 ℃, 95% CI − 0.31, − 0.06, p = 0.003).

**Conclusion:**

RBC variants were associated with non-severe malaria outcomes. Elucidation of the mechanisms by which they confer protection will improve understanding of genetic protection against malaria.

## Background

Multiple red blood cell (RBC) variants are associated with protection against severe *Plasmodium falciparum* malaria. Studies conducted in numerous populations have consistently shown that people with HbAS have a 70–90% reduced risk of severe malaria [1–4]. Similarly, both α−/αα and α−/α− thalassaemia reduce the risk of severe malaria by 20–40% [5–7]. The association between glucose-6-phosphate dehydrogenase [G6PD] deficiency and severe malaria is less clear [4, 8]. Some studies reported that G6PD protects both heterozygous females and hemizygous males from severe malaria [9], others reported protection for only hemizygous males [10], and others reported no protection [8]. Mechanisms through which these variants confer protection appear to be complex and may include restriction of red blood cell invasion and intracellular growth, increased destruction of parasitized red cells, and improved cell mediated and humoral immune responses [11–17].

While protective associations between certain RBC variants and severe malaria have been shown consistently [7, 18], associations with uncomplicated malaria have been less straightforward. Multiple studies showed reduced risk of malaria [19–24] and lower parasite densities [4, 25, 26] with HbAS compared to wild-type. For alpha thalassaemia, studies suggested both protection against and enhancement of malaria [5, 6, 27, 28]. For G6PD deficiency, studies in Africa [29–32], but not Asia [8, 33–35] suggested protection against uncomplicated malaria. Some discrepancies in findings might be attributed to differences between studies in human populations, transmission settings, and study designs.

Data from three cohorts conducted in areas of low, moderate, and high malaria transmission intensity in Uganda was used to quantify associations between three red blood cell variants and uncomplicated malaria outcomes. Notably, the cohort studies included passive surveillance for symptomatic malaria, active surveillance for *P. falciparum* infection, and regular entomological surveys to quantify transmission risk.

## Methods

### Study design, sites, and population

Prospective cohort studies were conducted at three sites. Study sites were Walukuba, Jinja District, a peri urban area in South Central Uganda with a low malaria transmission intensity (annual entomological inoculation rate (aEIR) or number of infective mosquito bites per person per year = 2.8); Kihihi, Kanungu District, a predominantly rural area in south western Uganda with moderate malaria transmission (aEIR = 32); and Nagongera, Tororo District, a predominantly rural area in south eastern Uganda with a very high transmission intensity (aEIR = 310) [36] until initiation of indoor residual spraying of insecticide in late 2014 [37]. Details on how the study households and participants were selected has been described elsewhere [36]. Briefly, in each of the 3 sub-counties 100 households were randomly enrolled. Households were included if they had at least one child between 6 months and 10 years of age and at least one adult resident providing informed consent. All children and one adult primary care giver from each household meeting the eligibility criteria were invited to participate. Participants were followed-up until they reached 11 years of age or until they were withdrawn from the study either voluntarily or because they failed to comply with study visits.

### Study procedures and follow-up

Study procedures and follow up have been described in detail [36–38]. Briefly, all participants were given an insecticide treated bed net at enrollment and were followed for all their healthcare needs at dedicated study clinics open 7 days each week. Participants were provided free health care and clinic travel expenses, but received no other incentives to participate. Episodes of malaria were diagnosed by passive case detection and defined as a history of fever within the past 24 h or an elevated temperature (≥ 38.0 ℃ tympanic) with a positive malaria thick blood smear. Episodes of malaria were treated with artemether-lumefantrine (uncomplicated malaria) or intravenous quinine or artesunate (complicated malaria), following national guidelines at the time of the studies. In addition, participants were asked to make a routine visit to the study clinic every 3 months. At each of these visits, a thick blood smear was evaluated to assess for parasitaemia. The cohorts were dynamic, such that all newly eligible children were enrolled, and participants were withdrawn when they reached 11 years of age.

Household level EIR was estimated using data from entomologic surveys carried out concurrently with the cohort studies, as previously described [39]. Briefly, one CDC light trap collection was carried out monthly in the main sleeping room of each house. The average household EIR was calculated as the average number of female *Anopheles* mosquitoes collected per night multiplied by the proportion of mosquitoes containing sporozoites at each site.

### Laboratory methods

Presence of three RBC variants, the sickle locus (rs334), − 3.7α− thalassemia and G6PD-G202A (rs1050828), was tested as previously described [40]. Briefly, genes of interest were amplified, amplicons subjected to mutation-specific restriction endonuclease digestion (for sickle haemoglobin and G6PD deficiency), reaction products resolved by electrophoresis, and genotypes determined based on the sizes of reaction products. For assessment of parasitaemia, thick blood smears were stained with 2% Giemsa, allowed to dry for 30 min, and read by experienced laboratory technologists. Parasite densities were calculated by counting the number of asexual parasites per 200 leukocytes or per 500 leukocytes if the count was less than 10 asexual parasites per 200 leukocytes, assuming a leukocyte count of 8000 per microlitre [41]. A blood smear was considered negative if the examination of 100 high power fields did not reveal any asexual parasites. For quality control, blood smears were read by a second microscopist, and discrepancies resolved by a third microscopist.

### Statistical methods

All data were collected using standardized forms and subsequently entered into Microsoft access before transfer to STATA (Version 14; STATA Corp., College Station, TX, USA) and R for analyses [42]. The data analysed were for participants enrolled from August 2011 through December 2014 and followed through June 2016. Study participants were included in the analyses if they had results for at least one RBC variant tested and at least 2 months of follow-up. Associations between RBC variants and malaria incidence were quantified using negative binomial regression models with measures of association expressed as the incidence rate ratio (IRR). Associations between RBC variants and parasite prevalence at the time of each routine visit were quantified using log-binomial generalized estimating equations with robust standard errors to generate prevalence ratios (PR). Effects of RBC variants on two components of anti-malarial immunity, anti-parasite immunity (ability to control parasite densities upon infection) and anti-disease immunity (ability to tolerate higher parasite density without developing fever) were also investigated. To model these outcomes, generalized additive models (GAMS) were used as previously described [38]. The outcome of interest in the anti-parasite immunity model was the log10 parasite density in parasite positive visits, and the outcome of interest in the anti-disease model was the measured temperature, adjusted for parasite density. All analyses were adjusted for age, average household level EIR, and study site, and included random effects at the individual and household level to account for clustering. For each RBC variant, the group with wild type genotype was chosen as the reference.

## Results

### Characteristics of participants in the study

**Fig. 1:**
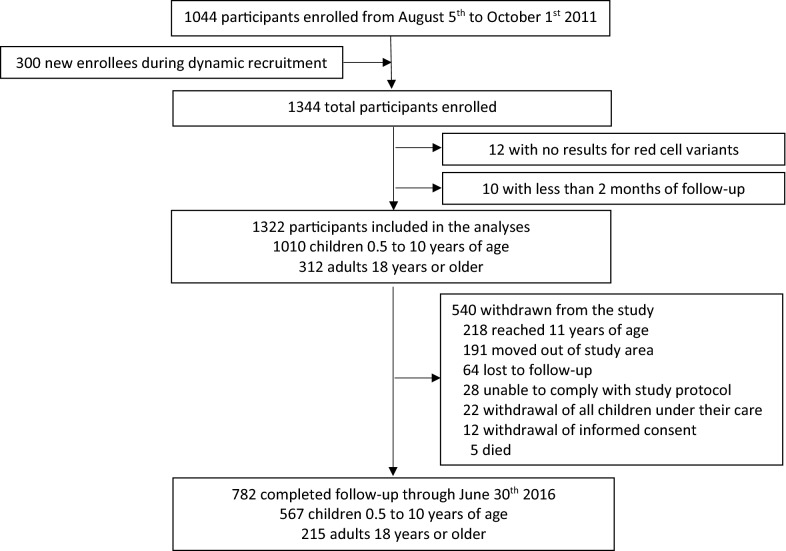
Flow diagram of participants included in the analyses

Of 1344 participants enrolled in the cohort studies, 1322 (98.4%) were included in the analyses and 782 of these (59.2%) were followed through the end of June 2016 (Fig. 1). A total of 1010 (76.4%) participants were children 0.5–10 years of age and 312 (23.6%) were adult primary care givers. Approximately half the children and 94% of adults were female (Table 1). Overall, the prevalence of all 3 red blood cell variants, Hb variant, alpha thalassaemia, and G6PD deficiency were highly variable across the three sites with Nagongera having the highest prevalence and Kihihi the lowest (Additional file 1: Table S1).

**Table 1:** Baseline characteristics of participants included in the analyses

Characteristic	Children, n (%)^a^	Adults, n (%)^a^
Study site		
Walukuba	318 (31.5%)	113 (36.2%)
Kihihi	357 (35.4%)	95 (30.5%)
Nagongera	335 (33.2%)	104 (33.3%)
Gender		
Male	516 (51.1%)	19 (6.1%)
Female	494 (48.9%)	293 (93.9%)
Haemoglobin variant		
AA	808 (80.7%)	242 (78.3%)
AS	188 (18.8%)	67 (21.7%)
SS	5 (0.5%)	0 (0%)
No result	9	3
Alpha thalassaemia variant		
Normal	585 (60.3%)	200 (66.0%)
α−/αα	335 (34.5%)	84 (27.7%)
α−/−α	50 (5.2%)	19 (6.3%)
No result	40	9
G6PD genotype (male)		
Normal	455 (89.4%)	15 (79.0)
Hemizygotes	54 (10.6%)	4 (21.1%)
No result	7	0
G6PD genotype (female)		
Normal	370 (75.5%)	232 (79.5%)
Heterozygotes	108 (22.0%)	55 (18.8%)
Homozygotes	12 (2.4%)	5 (1.7%)
No result	4	1

^a^Proportion among those with results available

### Associations between RBC variants and malaria incidence

The overall incidence of malaria across the 3 sites was 1.60 episodes per person year (PPY) among children and 0.31 episodes PPY among adults. Among children, HbAS was associated with a 22% lower incidence of malaria (IRR = 0.78, 95% CI 0.66–0.92, p = 0.003) and HbSS with an 83% lower incidence of malaria (IRR = 0.17, 95% CI 0.04–0.71, p = 0.02), compared to children with wild type genotypes (Table 2). In contrast, among adults there was no association between HbAS and malaria incidence (IRR = 0.99, 95% CI 0.66–1.49, p = 0.98). For both children and adults there were no significant associations between alpha thalassaemia or G6PD deficiency and malaria incidence (Table 2).

**Table 2:** Associations between RBC variants and malaria incidence

Age group	Red cell variants	Variant	Malaria episodes	Person years of follow-up	Incidence of malaria PPY	Unadjusted		Adjusted*	
						IRR (95% CI)	p-value	IRR (95% CI)	p-value
Children	Haemoglobin variant	AA	4612	2762	1.67	Reference		Reference	
		AS	818	620	1.32	0.77 (0.64–0.94)	0.009	0.78 (0.66–0.92)	0.003
		SS	3	13.4	0.22	0.16 (0.03–0.78)	0.02	0.17 (0.04–0.71)	0.02
	Alpha thalassaemia variant	Normal	3233	2061	1.57	Reference		Reference	
		α−/αα	1769	1106	1.60	1.00 (0.85–1.18)	0.98	1.04 (0.91–1.20)	0.56
		α−/−α	251	150	1.67	0.97 (0.68–1.38)	0.88	1.19 (0.87–1.62)	0.27
	G6PD genotype	Normal (male)	2680	1597	1.68	Reference		Reference	
		Hemizygotes	374	170	2.20	1.18 (0.85–1.63)	0.33	0.93 (0.71–1.22)	0.61
		Normal (female)	1768	1224	1.44	Reference		Reference	
		Heterozygotes	484	355	1.36	0.91 (0.70–1.18)	0.46	0.80 (0.63–1.01)	0.06
		Homozygotes	72	40.5	1.78	1.21 (0.60–2.44)	0.59	0.89 (0.51–1.57)	0.70
Adults	Haemoglobin variant	AA	300	936	0.32	Reference		Reference	
		AS	69	252	0.27	0.85 (0.56–1.30)	0.46	0.99 (0.66–1.49)	0.98
	Alpha thalassaemia variant	Normal	252	783	0.32	Reference		Reference	
		α−/αα	102	318	0.32	1.01 (0.68–1.48)	0.98	1.10 (0.76–1.59)	0.63
		α−/−α	11	70.0	0.16	0.50 (0.22–1.15)	0.10	0.58 (0.25–1.33)	0.20
	G6PD genotype	Normal (male)	13	55.1	0.23	Reference		Reference	
		Hemizygotes	3	19.4	0.16	0.66 (0.15–2.90)	0.58	0.98 (0.16–5.85)	0.98
		Normal (female)	294	995	0.30	Reference		Reference	
		Heterozygotes	73	191	0.38	1.26 (0.81–1.97)	0.31	1.13 (0.74–1.72)	0.58
		Homozygotes	1	11.2	0.09	0.39 (0.03–4.41)	0.45	0.40 (0.04–3.92)	0.43

* Adjusted for age, average household EIR, and study site

### Associations between RBC variants and prevalence of microscopic parasitaemia

The prevalence of microscopic parasitaemia at the time of routine visits across the 3 sites was 15.8% among children and 4.5% among adults. There were no significant associations between HbAS and parasite prevalence among children or adults. Children with HbSS had a 69% lower prevalence of parasitaemia compared to those with wild type genotype (PR = 0.31, 95% CI 0.18–0.54, p < 0.001) (Table 3). For both children and adults, α−/αα thalassaemia, but not α−/α− thalassaemia, was associated with a higher parasite prevalence compared to that in wild type individuals (RR = 1.23 95% CI 1.06–1.43, p = 0.008 and RR = 1.52, 95% CI 1.04–2.23, p = 0.03, respectively). For children and adults there were no significant associations between G6PD deficiency and microscopic parasitaemia (Table 3).

**Table 3:** Associations between RBC variants and prevalence of microscopic parasitaemia at the time of each routine visit

Age group	Red cell variants	Variant	Parasite prevalence	Unadjusted		Adjusted*	
				PR (95% CI)	p-value	PR (95% CI)	p-value
Children	Haemoglobin variant	AA	2488/15,830 (15.7%)	Reference		Reference	
		AS	597/3636 (16.4%)	1.07 (0.89–1.30)	0.46	0.95 (0.80–1.13)	0.57
		SS	3/66 (4.6%)	0.31 (0.14–0.68)	0.004	0.31 (0.18–0.54)	<0.001
	Alpha thalassaemia variant	Normal	1621/11,664 (13.9%)	Reference		Reference	
		alpha^+^ (α−/αα)	1272/6397 (19.9%)	1.45 (1.24–1.70)	<0.001	1.23 (1.06–1.43)	0.008
		alpha^0^ (α−/−α)	127/937 (13.6%)	0.96 (0.66–1.40)	0.83	0.98 (0.66–1.44)	0.91
	G6PD genotype	Normal (male)	1593/9147 (17.4%)	Reference		Reference	
		Hemizygotes	194/1030 (18.8%)	1.09 (0.81–1.48)	0.57	0.86 (0.65–1.14)	0.30
		Normal (female)	887/7039 (12.6%)	Reference		Reference	
		Heterozygotes	372/2062 (18.0%)	1.35 (1.04–1.75)	0.02	1.13 (0.87–1.45)	0.36
		Homozygotes	46/243 (18.9%)	1.38 (0.75–2.54)	0.30	0.92 (0.53–1.59)	0.76
Adults	Haemoglobin variant	AA	187/3952 (4.7%)	Reference		Reference	
		AS	37/1050 (3.5%)	0.72 (0.45–1.15)	0.17	0.74 (0.45–1.22)	0.24
	Alpha thalassaemia variant	Normal	128/3307 (3.9%)	Reference		Reference	
		alpha^+^ (α−/αα)	81/1332 (6.1%)	1.59 (1.08–2.34)	0.02	1.52 (1.04–2.23)	0.03
		α−/−α	15/290 (5.2%)	1.35 (0.76–2.42)	0.30	1.16 (0.63–2.14)	0.64
	G6PD genotype	Normal (male)	8/229 (3.5%)	reference		reference	
		Hemizygotes	7/79 (8.9%)	2.48 (0.72–8.52)	0.15	5.10 (0.58–44.9)	0.14
		Normal (female)	173/3792 (4.6%)	Reference		Reference	
		Heterozygotes	37/880 (4.2%)	0.87 (0.58–1.31)	0.51	0.77 (0.52–1.15)	0.20
		Homozygotes	3/50 (6.0%)	1.38 (0.42–4.50)	0.59	1.34 (0.44–4.05)	0.61

* Adjusted for age, average household EIR, and study site

### Associations between RBC variants and parasite densities upon infection (anti-parasite immunity) in children

Among children who were parasitaemic, the overall geometric mean parasite density was 11,400 parasites per µL (95% CI 10,800–12,100). On average, children with HbAS had lower parasite densities by a factor of 0.66 (95% CI 0.51–0.85, p = 0.001) compared to children with wild type genotype. There were no significant associations between alpha thalassaemia or G6PD deficiency and parasite densities (Table 4).

**Table 4:** Associations between red cell variants and parasite densities (anti-parasite immunity) in parasitaemic children

Red cell variants	Variant	Unadjusted		Adjusted*	
		Change in log^10^ parasite density (95% CI)	p-value	Change in log^10^ parasite density (95% CI)	p-value
Haemoglobin variant	AA	Reference		Reference	
	AS	− 0.25 (− 0.37, − 0.12)	< 0.001	− 0.18 (− 0.29, − 0.07)	0.001
	SS	− 0.4 (− 1.33, 0.53)	0.40	− 0.11 (− 0.95, 0.73)	0.80
Alpha thalassaemia variant	Normal	Reference		Reference	
	α−/αα	− 0.20 (− 0.30, − 0.10)	< 0.001	− 0.06 (− 0.14, 0.03)	0.19
	α−/−α	0.09 (− 0.14, 0.31)	0.45	0.04 (− 0.15, 0.22)	0.71
G6PD genotype	Normal (male)	Reference		Reference	
	Hemizygotes	− 0.01 (− 0.22, 0.20)	0.92	− 0.01 (− 0.18, 0.17)	0.94
	Normal (female)	Reference		Reference	
	Heterozygotes	− 0.18 (− 0.34, − 0.01)	0.04	− 0.05 (− 0.19, 0.09)	0.51
	Homozygotes	0.05 (− 0.34, 0.44)	0.80	0.12 (− 0.21, 0.45)	0.46

* Adjusted for age, average household EIR, and study site

### Associations between RBC variants and body temperature upon infection (anti-disease immunity) in children

Among children who were parasitaemic, the mean tympanic temperature was 37.6 °C (IQR 37.6–37.7). In models adjusted for parasite density, parasitaemic children with HbAS had body temperatures that were on average 0.13 °C lower (95% CI, 0.05–0.21, p = 0.002) at the time of the visit, compared to children with wild type genotype. There were no significant associations between alpha thalassaemia and body temperature among parasitaemic children. Upon stratification, parasitaemic male children that were G6PD hemizygotes had temperatures that were 0.19 °C lower (95% CI 0.06–0.31, p = 0.003) compared those with the wild type G6PD genotype. There were no significant associations between G6PD deficiency and temperature among female parasitaemic children (Table 5).

**Table 5:** Associations between RBC variants and temperature adjusted for parasite density (anti-disease immunity) in parasitaemic children

Red cell variants	Variant	Unadjusted		Adjusted*	
		Change in temperature in °C (95% CI)	p-value	Change in temperature in °C (95% CI)	p-value
Haemoglobin variant	AA	Reference		Reference	
	AS	− 0.13 (− 0.22, − 0.04)	0.003	− 0.13 (− 0.21, − 0.05)	0.002
	SS	0.09 (− 0.68, 0.86)	0.82	0.22 (− 0.52, 0.96)	0.56
Alpha thalassaemia variant	Normal	Reference		Reference	
	α−/αα	− 0.10 (− 0.17, − 0.03)	0.003	− 0.09 (− 0.21, 0.02)	0.11
	α−/−α	− 0.08 (− 0.24, 0.08)	0.31	− 0.16 (− 0.41, 0.08)	0.20
G6PD genotype	Normal (male)	Reference		Reference	
	Hemizygotes	− 0.12 (− 0.25, 0.01)	0.07	− 0.19 (− 0.31, − 0.06)	0.003
	Normal (female)	Reference		Reference	
	Heterozygotes	− 0.12 (− 0.24, 0.01)	0.07	− 0.09 (− 0.21, 0.02)	0.11
	Homozygotes	− 0.08 (− 0.37, 0.20)	0.57	− 0.16 (− 0.41, 0.08)	0.20

* Adjusted for parasite density, age, average household EIR, and study site

## Discussion

This study used data from 1322 participants enrolled in three longitudinal cohorts in Uganda to investigate associations between RBC variants and several outcomes of uncomplicated *P. falciparum infection*. Compared to children with wild type haemoglobin, children with HbAS had a reduced risk of incident malaria, developed lower parasite densities upon infection, and tolerated higher parasite densities without developing fever. Associations with other RBC variants were less straightforward: α−/αα thalassaemia was associated with higher parasite prevalence in both children and adults; G6PD deficient male hemizygote children, but not females, tolerated higher parasite densities without developing fever. These findings are consistent with recent findings from Uganda and elsewhere showing that HbAS is associated with protection in children, but not adults, from symptomatic malaria [2, 7, 19] and is also associated with low parasite densities during symptomatic disease [4, 19, 43]. Children with HbSS were also strongly protected against incident malaria. Furthermore, HbSS children were also less likely to develop high temperatures for any given parasite density, as compared to children with wild type haemoglobin (i.e.; they had higher anti-disease immunity). The mechanisms by which HbAS protects the host against symptomatic and severe disease have been extensively explored and appear to be complex. Some of the proposed mechanisms include increased sickling of parasitized RBCs, enhanced phagocytosis of parasitized RBCs, reduced intraerythrocytic parasite growth, and decreased cytoadherence via reduced expression of *P. falciparum* Erythrocyte Membrane Protein 1 (PfEMP-1) [11, 44]. The lack of protection associated with HbAS in adults is likely due to the predominant role played by acquired immunity among older age groups in highly endemic regions.

In contrast to results for HbS, there were no protective associations between alpha thalassaemia and any of the outcomes measured. In fact, although α−/αα thalassaemia has been associated with protection against both severe [5, 6, 18, 22] and uncomplicated forms of malaria [27, 28] in this study of uncomplicated malaria it was associated with a higher parasite prevalence in both children and adults as compared to the wild type. Similar associations have been previously suggested [45]. However, α−/αα thalassaemia did not have any association with incidence of symptomatic disease, parasite densities or anti-disease immunity.

Interestingly, the results from this study suggest that G6PD deficiency may play a role in anti-disease immunity. For any given parasite density, G6PD hemizygous parasitaemic male children had lower temperatures than wild type parasitaemic male children upon *P. falciparum* infection. Thus, G6PD deficiency may be associated with increased ability to tolerate high parasite densities without developing fever. However, the protective effect against symptomatic malaria was seen only in males, and not in heterozygous or homozygous females. There is considerable evidence to suggest that G6PD protects both male [10] and female [30, 46, 47] African children against severe malaria. However, information regarding G6PD protection from uncomplicated malaria is less clear albeit with a leaning towards protection for female children [4, 8, 24].

Results for associations between G6PD deficiency and uncomplicated malaria remain inconclusive.

This study had several limitations. First, sample sizes were small for certain groups, notably the SS and G6PD homozygous genotypes, which limited the ability to evaluate their associations with malaria outcomes. However, these genotypes occurred at expected frequencies in these populations and sample sizes would be prohibitively large to study them in a cohort. Second, parasitaemia was measured using microscopy, which is much less sensitive than molecular techniques for identifying parasites [48–50]. Impacts of RBC variants on sub-microscopic parasitaemia were not studied. Third, the degree of fever measured during symptomatic malaria may have been influenced by children receiving antipyretics prior to evaluation in the clinic. However, the majority of the patients presented to the study clinics within 24–48 h of symptom onset. Moreover, participants were encouraged to present to the clinic for all their health care needs as soon as they were unwell. Fourth, the study did not explore associations between red blood cell variants and other measures of protective immunity such as *P. falciparum* antibodies. Finally, there were very few cases of severe malaria, precluding exploration of associations between host polymorphisms and severe malaria.

## Conclusion

In areas endemic for falciparum malaria, RBC variants may play an important role in protecting children against malaria. First, both HbAS and alpha thalassaemia are associated with decreased incidence of severe malaria. These strong associations probably explain the persistence of these balanced polymorphisms in human populations at risk of falciparum malaria. Second, some polymorphisms also appear to protect against uncomplicated malaria. Identified associations with HbAS provide the strongest evidence of protection, with evidence for lower risks of malarial incidence and enhancement of both anti-parasite and anti-disease immunity. G6PD deficiency may provide additional protection against progression to symptomatic disease, with evidence for enhanced anti-disease immunity. While the mechanisms by which common RBC variants protect against malaria remain incompletely understood, their associations with protection against malaria underline the exceptional selective pressure of malaria on the human genome.

## Supplementary information

**Additional file 1: Table S1.** Prevalence of red blood cell variants stratified by study site and age group.

## Data Availability

All data has been made publicly available at https://clinepidb.org/ce/app.

## Abbreviations

G6PD: glucose-6-phosphate dehydrogenase
HbAS: haemoglobin AS genotype
HbSS: haemoglobin SS genotype
HbS: haemoglobin S genotype
PCR: polymerase chain reaction
LAMP: loop mediated isothermal amplification
GAMS: generalized additive models
aEIR: annual entomological inoculation rate
