# Supplementary material for: Associations between red blood cell variants and malaria among children and adults from three areas of Uganda: a prospective cohort study
Source: Malar J. 2020 Jan 15;19:21. doi: 10.1186/s12936-020-3105-3 (PMC6964006; doi:10.1186/s12936-020-3105-3)
Supplement: Supplementary file 1 — Additional file 1: Table S1. Prevalence of red blood cell variants stratified by study site and age group. [file 12936_2020_3105_MOESM1_ESM.docx]

**Additional file 1: Table S1. Prevalence of red blood cell variants stratified by study site and age group**

| **Red blood cell variants** | | **Children, n (%*)** | | | **Adults, n (%*)** | | |
| --- | --- | --- | --- | --- | --- | --- | --- |
|  |  | **Walukuba** | **Kihihi** | **Nagongera** | **Walukuba** | **Kihihi** | **Nagongera** |
| Haemoglobin  variant | AA | 241 (76.3%) | 332 (93.3%) | 235 (71.4%) | 77 (69.4%) | 88 (92.6%) | 77 (74.8%) |
|  | AS | 72 (22.8%) | 24 (6.7%) | 92 (28.0%) | 34 (30.6%) | 7 (7.4%) | 26 (25.2%) |
|  | SS | 3 (0.9%) | 0 (0%) | 2 (0.6%) | 0 (0%) | 0 (%) | 0 (%) |
|  | No result | 2 | 1 | 6 | 2 | 0 | 1 |
| Alpha thalassaemia variant | Normal | 167 (52.8%) | 275 (80.9%) | 143 (45.5%) | 68 (60.7%) | 80 (86.0%) | 52 (53.1%) |
|  | α-/αα | 120 (38.0%) | 61 (17.9%) | 154 (49.0%) | 36 (32.1%) | 13 (14.0%) | 35 (35.7%) |
|  | α-/-α | 29 (9.2%) | 4 (1.2%) | 17 (5.4%) | 8 (7.1%) | 0 (0%) | 11 (11.2%) |
|  | No result | 2 | 17 | 21 | 1 | 2 | 6 |
| G6PD genotype (male) | Normal | 144 (90.6%) | 163 (96.4%) | 148 (81.8%) | 7 (100%) | 4 (100%) | 4 (50.0%) |
|  | Hemizygotes | 15 (9.4%) | 6 (3.6%) | 33 (18.2%) | 0 (0%) | 0 (0%) | 4 (50.0%) |
|  | No result | 2 | 3 | 2 | 0 | 0 | 0 |
| G6PD genotype (female) | Normal | 114 (73.1%) | 161 (88.0%) | 95 (62.9%) | 88 (83.0%) | 83 (92.2%) | 61 (63.5%) |
|  | Heterozygotes | 40 (25.6%) | 21 (11.5%) | 47 (31.1%) | 16 (15.1%) | 7 (7.8%) | 32 (33.3%) |
|  | Homozygotes | 2 (1.3%) | 1 (0.5%) | 9 (6.0%) | 2 (1.9%) | 0 (0%) | 3 (3.1%) |
|  | No result | 1 | 2 | 1 | 0 | 1 | 0 |

***** Proportion among those with results available
